# Association between malnutrition and anemia in under-five children and women of reproductive age: Evidence from Bangladesh Demographic and Health Survey 2011

**DOI:** 10.1371/journal.pone.0219170

**Published:** 2019-07-03

**Authors:** M. Shafiqur Rahman, Muntaha Mushfiquee, Mohammad Shahed Masud, Tamanna Howlader

**Affiliations:** Institute of Statistical Research and Training, University of Dhaka, Dhaka, Bangladesh; University of Ghana, GHANA

## Abstract

**Background:**

Bangladesh is one of the most anemia prone countries in South Asia. Children of age under five years and women of reproductive age are particularly vulnerable in this region. Although several studies have investigated the risk factors of anemia, only few have explored its association with malnutrition, despite its high prevalence in the same group. The objective of this paper is to investigate the association of malnutrition with anemia by conducting separate analyses for under-five children and women of reproductive age using data from the nationally representative 2011 Bangladesh Demographic and Health Survey.

**Methods:**

Two binary outcome variables are considered separately: presence of anemia in children under five years of age (Hb<11.0 g/dl) and presence of anemia in women of childbearing age (Hb<12.0 g/dl). The exposures of interest corresponding to these two outcomes are stunting (low height-for-age) and low BMI (<18.5 kg/m^2^), respectively. Preliminary analysis involves estimating the association between exposure and outcome while controlling for a single confounder by computing adjusted odds ratios (adjOR) using the Cochran-Mantel-Haenszel approach in stratified analysis. Later, associations between the exposures and outcomes are estimated separately for under-five children and women of reproductive age by fitting multivariable regression models that adjust simultaneously for several confounders.

**Results:**

The prevalence of anemia is found to be higher among both the stunted children and women with low BMI compared to their healthy counterparts (Children: 56% vs 48%; women: 50% vs 43%). Furthermore, stunted children and women with low BMI have significantly increased odds of developing anemia, as reflected by the adjusted ORs of 1.76 (95% CI:1.10–2.83) and 1.81 (95% CI: 1.11–3.48), respectively. The association of stunting with anemia in children was modified by their age and socio-economic condition, where risk of being anemic decreases with increasing age but with a lower rate for stunted children from richest family. In addition, stunted children of anemic mothers are at greater risk of being anemic compared to non-stunted children of anemic or non-anemic mothers. Again the association between BMI and anemia in women is modified by the level of education, with risk of anemia being lowest among women with low BMI and higher education.

**Conclusion:**

Evidence–based policies targeting the vulnerable groups are required to combat anemia and nutritional deficiencies simultaneously under the same program.

## Introduction

Anemia, which is characterized by low level of hemoglobin in the blood, is one of the major public health hazards affecting people in both developed and developing countries [1–3]. Anemia may occur at all stages of life, however, young children and women in the childbearing age are the most vulnerable [4, 5]. When anemia occurs in children, it could affect their cognitive performance and physical growth [6]. In women, anemia could adversely affect their capacity to work and may lead to poor pregnancy outcomes [7]. According to the World Health Organization (WHO), globally about 38% of women of reproductive age and 43% of children under five years of age were affected by anemia in 2011 [2, 3]. Anemia is more prevalent in developing countries [4, 8] contributing to about one million deaths each year world-wide. Three-quarters of these deaths occur in Africa and South-East Asia [2, 3, 9, 10]. Bangladesh has been reported as one of the most anemia prone countries in South Asia [11–13]. According to the National Nutrition Project (NNP), the prevalence of anemia among children of ages 6–59 months was estimated to be 47% in 2004 and 68% in 2013 [14, 15]. The National Micronutrient Survey 2011–12 estimated an anemia prevalence of 33% among the children of the age group 6–59 months and 26% among the non-pregnant and non-lactating women [14]. Another study reported that childhood anemia decreased with increasing age, with a prevalence of 64% among children of ages 6–23 months and 42% among children of ages 24–59 months [15, 16]. Such high prevalence reported by multiple studies indicates that anemia is a major public health threat in Bangladesh. Although Bangladesh has made remarkable progress in health and social development achieving most of the Millenium Development Goals (MDGs) in the last decade [17], it is still struggling to tackle the burden of some diseases including anemia.

Several studies [1, 18–21] have been conducted to identify the factors associated with anemia among the vulnerable groups of the population. Most of these studies reported iron deficiency as the primary cause of anemia in developing countries along with other associated causes including malaria, parasitic infection, nutritional deficiencies, and haemoglobinpathies (a genetic condition). However, recent evidence suggests that iron deficiency may not be the primary cause of anemia in Bangladesh [22, 23]. The reason is that iron is abundant in groundwater and majority of the country’s population relies on groundwater for drinking purposes [24]. Thus consumption of iron occurs through consumption of groundwater. Furthermore, there are studies that have found a link between the amount of iron intake through groundwater and the level of iron in the body [23, 25]. Thus, iron deficiency does not appear to be the most important risk factor of anemia, which explains why most of the iron-supplementation intervention programmes are ineffective in reducing the burden of anemia in Bangladesh [26].

Given the low prevalence of iron deficiency but high prevalence of anemia among women and children in Bangladesh, some recent studies in similar [27, 28] or different socio-economic settings [29–31] were conducted to examine whether socio-demographic factors are associated with anemia. Most of these studies identified household socioeconomic status, food insecurity, and geographical location as risk factors. Although some of these studies identified stunting and low body-mass-index (BMI) as one of the risk factors of anemia in under-five children and women of reproductive age, respectively, limited studies have explicitly explored the association of these malnutrition indicators with anemia, despite their high prevalence in both women and children. According to recent evidence from the national surveys and studies [32, 33], about 36% of under-five children are stunted (low height-for-age), 33% are underweight (low weight-for-age) and 14% are wasted (low weight- for-height). Similarly 19% of women of reproductive age are malnourished (low BMI against the standard level 18.5). Thus, women of reproductive age and under-five children in Bangladesh are also vulnerable to malnutrition [32, 34]. High prevalence of anemia and malnutrition in both populations hints at a possible link between these two conditions. Thus, an intensive investigation is required to determine how and to what extent the nutritional statuses of women and children are associated with their anemia levels and whether the association is modified by other risk factors. Although some studies have [35, 36] tried to identify common factors associated with anemia and growth (measured by stunting, underweight and wasting in children and low BMI in women) through separate analysis of anemia and growth data, such an analysis may not be useful in identifying whether there is any association between growth and anemia. Against this backdrop, the current paper examines the association between anemia and stunting in under-five children and the association between anemia and BMI in childbearing women using nationally representative data extracted from the Bangladesh Demographic and Health Survey 2011. Findings of the study will be useful in providing new insights that may help design effective policies for reducing the burden of anemia as well as malnutrion in both women and children.

## Methods

### Data

Data on anemia, stunting and BMI were extracted from the 2011 Bangladesh Demographic and Health Survey (BDHS) [37] conducted during November 2010- March 2011. BDHS is a nationally representative health survey conducted every three years since 1993 with collaborative efforts of the National Institute of Population Research and Training (NIPORT), ICF International (USA), and Mitra and Associates under the demographic and health survey (DHS) program based on developing countries across the world. It is a cross-sectional study based on a two-stage stratified cluster sampling design. The entire population was stratified into 14 strata based on 7 administrative divisions and urban-rural areas in each division. In the first stage, the primary sampling units (PSUs) consisting of ward in rural area or sub-ward in urban area were randomly selected from a list of PSUs in each stratum. An equal number of households were then randomly selected from each PSU in the second stage. The survey collected information on health, nutrition and demographic history for men, women and children. In particular, data on anemia were collected from women of reproductive age and children in the age group 6–59 months belonging to every third household in the selected sampled households by screening the hemoglobin(Hb) levels (g/dl) in their blood samples at the time of survey. The 2011 BDHS used HemoCue^®^Hb 201^+^ rapid testing methodology, which consists of a battery-operated photometer and a disposible microcuvatte to measure Hb levels in the blood sample (a drop of capillary blood taken from fingertip). The classification of women and children as anemic or non-anemic was performed after adjusting their Hb levels for altitude, and in the case of women, pregnancy status was also adjusted for using Centre for Disease Control (CDC) formula [38]. Information on stunting and BMI were also collected at the time of the survey by taking anthropometric measurments such as height, weight and age of the women and children. For more details on survey methodology and methods for measurement of anemia, see elsewhere [37].

### Ethical considerations

The Ethics committee at NIPORT, Mitra and Associates, and ICF international approved a waiver from ethical approval for this retrospective study. As the de-identified data for this study came from the secondary sources, this study does not require eithical approval.

### Variables

The present study considered two populations, i.e., under-five children (6–59 months) and women of reproductive age (15–49 years) and therefore separate outcome-exposure pairs for the two populations. The outcome variables were ‘maternal-anemia’ (yes, no) representing anemia status in women and ‘child-anemia’ (yes, no) representing anemia status in children. Children were classified as anemic if their adjusted hemoglobin levels (Hb) were less than the cuttoff of 11.0 g/dl, and women were classified as anemic if Hb<12.0g/dl. The exposure variables of interest were BMI in the case of women and stunting in the case of children. BMI is a quantitative variable that was calculated using height and weight measurements of women and is a commonly used nutritional status indicator. BMI was convereted to a binary exposure with categories low (BMI<18.5) and high (BMI ≥18.5). Thus, women were considered malnourished if their BMI was less than 18.5. Similarly stunting (low height for age), which is a good nutritional status indicator for children, was also converted to a binary exposure (stunted vs normal). Children having z-score for the height-for-age index less than two standard deviations from the median of WHO reference population [37] were considered as stunted.

In addition to the above exposures, a set of background factors were chosen based on previous literature to control for potential confounders of the association between the exposure and outcome [27, 39]. Tables 1 and 2 list the qualitative background risk factors and their corresponding levels/categories for the outcomes child anemia and maternal anemia, respectively. In Table 1, a child was considered to have access to food if he/she had three square meals per day regularly. Otherwise, the child had limited access. In Table 2, information on size at birth was collected retrospectively by asking mothers to recall whether the child’s size was ‘very small’, ‘smaller than average’, ‘average’, or ‘above average’ at birth. This information is commonly used as proxy for birth weight in many studies [40, 41] as majority of births in Bangladesh occur at home where there is usually no facility for taking the baby’s weight. The binary risk factor ‘size at birth’ was created by classifying a child as ‘small’ if the mother reported ‘very small’ or ‘smaller than average’. Otherwise, the child’s size was considered normal. The variable socio-economic status (SES) was determined from wealth index that was calculated by performing principal component analysis on the assests owned by the households. The first principal component, which explains maximum variability of the data, gives the wealth index. The wealth index was then used to categorize the individuals into five equal groups, where the first quintile refers to the poorest group and the fifth quintile refers to the richest group [37].

**Table 1:** Distribution of anemic children by their background characteristics.

Variables	Number at risk	% of anemic children	Variables2	Number at risk	% of aanemic children
**Stunting**			**Maternal anemia status**		
Stunted	925	56.5	not anemic	1239	45
Normal	1309	48.5	anemic	1000	61.3
**Sex**			**Size at birth**		
Male	1173	53.1	Normal	1883	52.3
Female	1110	51.1	Small	399	51.6
**Age in months**			**Birth order**		
24-Jun	780	71	First	791	52.2
25–48	1019	44.5	2–3	1020	51.5
48–59	484	38	4+	472	53.6
**Maternal Education**			**Child had fever recently**		
No education	432	53.5	no	1366	49.6
Primary	744	54.6	Yes	917	56.1
Secondary	947	52.1	**Child had diarrhea recently**		
Higher	160	38.1	no	2154	51.8
**SES**			Yes	129	58.1
Poorest	521	59.5	**Received Vitamin A supplement**		
Poorer	445	58	Yes	531	58
Middle	408	52.4	no	1740	50.3
Richer	431	45.7	**Access to food**		
Richest	408	44	Yes	1830	51.3
**Region**			Limited	453	55.6
Barisal	256	59.4	**Area of residence**		
Chittagong	437	52.2	Urban	686	48.3
Dhaka	370	47.8	rural	1597	53.8
Khulna	254	54.3	**Total**	2271	52.1
Rajshahi	281	48.4			
Rangpur	305	57.7			
Sylhet	380	48.4			

**Table 2:** Distribution of anemic women by their background characteristics.

Variables	Number at risk	% of anemic women
**BMI**		
BMI<18.5	719	49.5
BMI> = 18.5	1739	43.2
**Births in last five years**		
1	1713	42.6
2–4	754	50.5
**Age in years**		
15–19	307	46.3
20–29	1530	43.5
30+	628	48.3
**Education**		
No education	486	51.6
Primary	798	47.2
Secondary	1010	41.4
Higher	173	38.2
**SES**		
Poorest	562	51.6
Poorer	494	50.4
Middle	458	47.2
Richer	457	41.6
Richest	496	33.7
**Region**		
Barisal	270	48.5
Chittagong	465	41.7
Dhaka	404	49.7
Khulna	274	35.8
Rajshahi	313	45.7
Rangpur	328	48.8
Sylhet	413	44.8
**Area of residence**		
Urban	747	41.2
Rural	1720	46.7
**Access to food**		
Yes	1977	43.5
Limited	490	51.2

### Statistical analysis

First, univariate analysis was performed by calculating descriptive statistics that were used to summarize the data. Next, bivariate analysis was performed by calculating odds ratios from 2x2 contigency tables for the exposure and outcome variables. However, the true association between stunting and child-anemia or BMI and maternal-anemia may be confounded by other risk factors known as confounders that are associated with both the outcome and exposure of interest. To mitigate the effects of confounders, stratified analysis was performed by constructing 2x2 contigency tables for the exposure and outcome at each level of the confounder and computing the stratum specific odds ratios (OR). These odds ratios are then combined using Cochran-Mantel-Haenszel approach [42], which takes the weighted average of the strafum specific odds ratios to obtain the adjusted odds ratio (adjOR). The stratum specific analyses are also useful for identifying potential confounders and effect modifiers thereby providing insights for building a good multivariable regression model. Following stratified analysis, the net effects of malnutrition on child-anemia and maternal-anemia were assessed separately while controlling for the effects of all confounders simultaneously by fitting multivariable logistic models containing both main and interaction effects. Although several confounders were identified during the bivariate analysis, the final model contained the main and interaction effects of only a few confounders. The other confounders were excluded one by one based on the p-value (>0.10) of the likelihood ratio-test performed by adding the confounder to the model starting with stunting (for the model with child anemia) and BMI (for model with maternal anemia) in different combinations either as main effects and/or interaction effect. The non-linearity of the continuous covariate was assessed by introducing a quadratic term for the covariate and observing whether it is significant. If the quadratic term was significant, it was retained along with the linear term to capture the non—linear effect of the covariate on the outcome. Each estimate from the above analysis including the model fitting was obtained by employing sampling weight. All statistical analyses were performed using combination of Stata package ‘svy’, svyset, ‘epitab’, and ‘logit’ in Stata version 14.

## Results

The analysis was based on a sample of 2283 children of ages between 6–59 months (male 41.4% and female 58.6%) and 2467 women of ages between 15–49 years. The average age of the children was 33.03 months with standard deviation (SD) of 15.86 months (results not shown). More than two-fifth of the children (41.4%) were stunted. Fifty one percent of children (n = 2234) with ages between 6 to 59 months were estimated to be anemic. The average age of the women in the sample was 25.91 years with SD of 5.97 years. The percentage of women of reproductive age having BMI<18.5 was 29.25%. Forty five percent of the women (n = 2467) were found to be affected by anemia. Table 1 presents the percentage of anemic children in the different categories of the background risk factors. Results indicate that the prevalence of anemia is higher among stunted children than among normal children (56.5% vs 48.5%). The prevalence of anemia is higher among women with low BMI (<18.5) than among women with normal BMI (>18.5) (49.5% vs 43.2%). Furthermore, prevalence of anemia is found to be higher among stunted children compared to non-stunted children and among women with low BMI compared to those with normal BMI at each level of the confounder in stratified analysis (Tables 3 and 4).

**Table 3:** Association between stunting and anemia in children of age 6–59 months.

Confounder Categories	Nutritional Status	Number at risk	% anemic child	OR (95%CI)	adj OR (95% CI)
**Sex of child**					
Male	stunted	465	58.5	1.47(1.16–1.87)	1.39(1.18–1.66)
	normal	679	48.9		
Female	stunted	465	54.8	1.32(1.04–1.68)	
	normal	625	47.8		
***Age of a child***					
6 to 24	stunted	294	75.2	1.42(1.02–1.97)	1.50(1.26–1.79)
	normal	467	68.1		
25 to 48	stunted	447	49.2	1.46(1.13–1.87)	
	normal	553	39.9		
48 to 59	stunted	189	45.5	1.74(1.19–2.54)	
***Size of a child***					
Normal size	stunted	732	56.0	1.32(1.09–1.59)	
	normal	1115	49.2		
Below average	stunted	198	59.1	1.87(1.25–2.79)	
***Birth order***					
First	stunted	308	56.5	1.35(1.01–1.80)	1.39(1.17–1.65)
	normal	463	49.0		
2 to 3	stunted	383	54.3	1.24(.96–1.60)	
	normal	619	48.9		
4+	stunted	239	60.7	1.85(1.28–1.67)	
	normal	222	45.5		
***Child recently had diarrhea***					
No	stunted	873	56.6	1.42(1.19–1.69)	1.39(1.18–1.65)
	normal	1234	47.9		
Yes	stunted	57	57.9	1.03(.51–2.08)	
	normal	70	57.1		
***Child had fever recently***					
No	stunted	544	54.6	1.44(1.16–1.79)	1.33(1.17–1.64)
	normal	795	45.5		
Yes	stunted	386	59.6	1.32(1.00–1.72)	
	normal	509	52.6		
***Currently breast feeding***					
No	stunted	306	47.1	1.61(1.20–2.16)	1.37(1.15–1.62)
	normal	481	35.6		
Yes	stunted	624	61.4	1.25(1.01–1.55)	
	Normal	823	55.9		
***Mother’s education***					
No education	stunted	232	56.5	1.34(.91–1.97)	1.34(1.13–1.59)
	normal	189	49.2		
Primary	stunted	363	55.1	1.07(.79–1.43)	
	normal	365	53.4		
Secondary	stunted	304	59.9	1.63(1.23–2.15)	
	normal	623	47.8		
Higher	stunted	31	45.2	1.50(.69–3.29)	
	normal	127	35.4		
***SES***					
Poorest	stunted	295	58.3	.91(.63–1.29)	1.26(1.06–1.50)
	normal	216	60.7		
Poorer	stunted	210	60.0	1.22(.84–1.78)	
	normal	234	55.1		
Middle	stunted	161	52.8	1.06(.71–1.59)	
	normal	238	51.3		
Richer	stunted	149	53.7	1.66(1.11–2.48)	
	normal	272	41.2		
Richest	stunted	115	55.7	1.89(1.24–2.90)	
	normal	344	39.8		
***Region***					
Barisal	stunted	112	62.5	1.34(.80–2.22)	1.40(1.18–1.67)
	normal	137	55.5		
Chittagong	stunted	185	58.4	1.60(1.09–2.36)	
	normal	238	46.6		
Dhaka	stunted	158	47.5	1.00(.66–1.52)	
	normal	205	47.3		
Khulna	stunted	86	63.9	1.75(1.02–2.99)	
	normal	165	50.3		
Rajshahi	stunted	99	55.6	1.60(.98–2.62)	
	normal	178	43.8		
Rangpur	stunted	122	63.1	1.48(.92–2.36)	
	normal	177	53.4		
Sylhet	stunted	168	51.8	1.33(.89–2.00)	
	normal	204	44.6		
***Type of place of residence***					
Urban	stunted	247	59.5	2.09(1.52–2.87)	1.38(1.16–1.63)
	normal	426	41.3		
Rural	stunted	683	55.6	1.17(.95–1.43)	
	normal	878	51.8		
Overall	stunted	925	56.5	1.38(1.17, 1.63)	
	normal	1309	48.5		

When the strength of the association between anemia and stunting in children, or anemia and BMI in women was quantified by calculating the stratum-specific odds ratio at each level of the confounder, strong and significant associations were observed in each stratum. Tables 3 and 4 also show the adjOR, which measures the association between anemia and the exposure variable (stunting or BMI) after adjusting for the confounder and are calculated using the Cochran-Mantel-Haenszel approach. One observes that the associations remain significant even after adjusting for the effect of the confounder. For example, after controlling for the effect of sex, stunted children had 39% greater odds of developing anemia than normal children with estimated adjOR of 1.39 (95% CI: 1.18–1.66) **(**Table 3). Higher odds of being anemic among stunted children compared to non-stunted children were also observed after controlling for each of the other confounders, such as, age, SES etc, separately. Again, women with low BMI (BMI<18.5) had 29% greater odds of having anemia than women with normal BMI [adjOR = 1.29 (95% CI: 1.08–1.53)], after controlling for the effect of age **(**Table 4). Similar findings were observed when controlling for the other confounders such as age, education, and SES etc, separately.

**Table 4:** Association between BMI and anemia in women of age 15–49 years.

Confounder categories	BMI	Numbers’ at risk	% of anemic women	OR (95%CI)	adjOR (95%CI)
**Mother’s age**					
15 to 19	BMI<18.5	153	51.6	1.36(.89–2.07)	1.29(1.08–1.53)
	BMI> = 18.5	214	43.9		
20 to 29	BMI<18.5	428	48.1	1.25(1.00–1.56)	
	BMI> = 18.5	1187	42.6		
30 to 39	BMI<18.5	144	55.6	1.35(.93–1.98)	
	BMI> = 18.5	425	48		
40 to 49	BMI<18.5	24	50.0	1.19(.45–3.16)	
	BMI> = 18.5	46	45.7		
**Food**					
Access to food	BMI<18.5	549	47.2	1.17(.96–1.42)	1.25(1.05–1.48)
	BMI> = 18.5	1565	43.3		
Limited access to food	BMI<18.5	200	59	1.55(1.08–2.21)	
	BMI> = 18.5	307	48.2		
**Birth in last 5 year**					
1	BMI<18.5	472	46.0	1.17(.95–1.44)	1.25(1.06–1.49)
	BMI> = 18.5	1339	42.1		
2–4	BMI<18.5	277	57.8	1.43(1.06–1.91)	
	BMI> = 18.5	533	48.9		
**SES**					
Poorest	BMI<18.5	242	55.4	1.12(.81–1.56)	1.13(.95–1.35)
	BMI> = 18.5	343	52.5		
Poorer	BMI<18.5	183	56.3	1.48(1.03–2.13)	
	BMI> = 18.5	329	47.1		
Middle	BMI<18.5	144	48.6	1.09(.75–1.62)	
	BMI> = 18.5	350	46.3		
Richer	BMI<18.5	119	45.4	1.20(.79–1.81)	
	BMI> = 18.5	376	40.9		
Richest	BMI<18.5	61	24.6	.56(.31–1.03)	
	BMI> = 18.5	474	36.7		
**Region**					
Barisal	BMI<18.5	79	50.6	1.06(.63–1.78)	1.26(1.06–1.49)
	BMI> = 18.5	193	49.2		
Chittagong	BMI<18.5	123	48.0	1.38(.92–2.08)	
	BMI> = 18.5	390	40.0		
Dhaka	BMI<18.5	125	58.4	1.60(1.05–2.43)	
	BMI> = 18.5	295	46.9		
Khulna	BMI<18.5	66	40.9	1.23(.71–2.15)	
	BMI> = 18.5	228	36.0		
Rajshahi	BMI<18.5	84	45.2	.98(.60–1.61)	
	BMI> = 18.5	243	45.7		
Rangpur	BMI<18.5	113	52.2	1.06(.68–1.65)	
	BMI> = 18.5	281	42.7		
**Type of place of residence**					
Urban	BMI<18.5	160	46.8	1.32(.94–1.88)	1.25(1.05–1.48)
	BMI> = 18.5	641	39.9		
Rural	BMI<18.5	589	51.3	1.22(1.00–1.49)	
	BMI> = 18.5	1231	46.2		
**Mother’s education**					
No education	BMI<18.5	172	60.5	1.66(1.15–2.42)	1.22(1.03–1.45)
	BMI> = 18.5	330	47.9		
Primary	BMI<18.5	282	52.5	1.24(.93–1.66)	
	BMI> = 18.5	527	42.1		
Secondary	BMI<18.5	279	42.7	1.04(.79–1.36)	
	BMI> = 18.5	842	41.8		
Higher	BMI<18.5	16	37.5	.95(.34–2.64)	
	BMI> = 18.5	173	38.7		
**Overall**	BMI<18.5	719	49.5	1.29 (1.08, 1.53)	
	BMI> = 18.5	1739	43.2		

**Fig 1:**
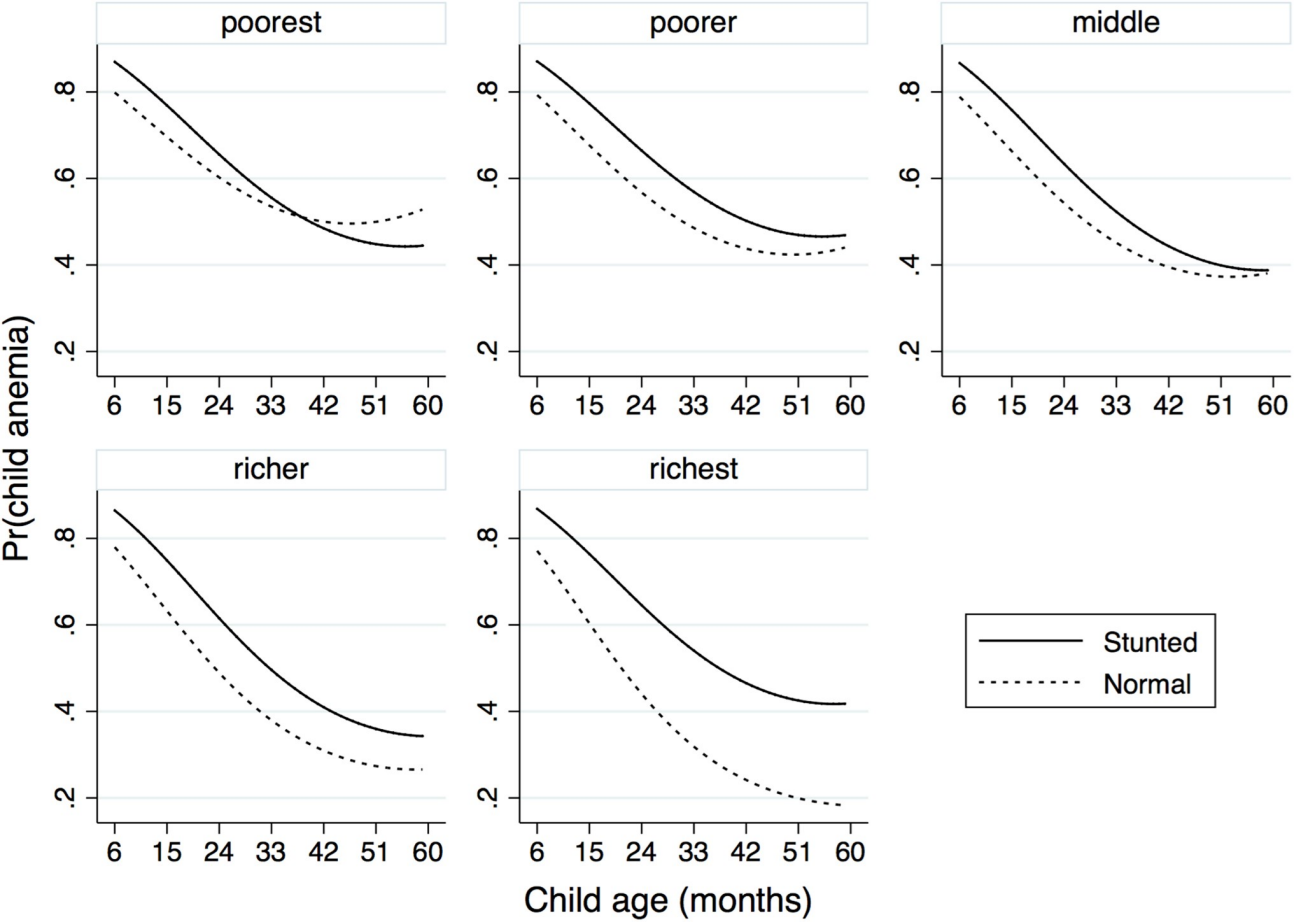
Interaction plot describing the effect of interaction between stunting, SES and age on the risk of child anemia.

**Fig 2:**
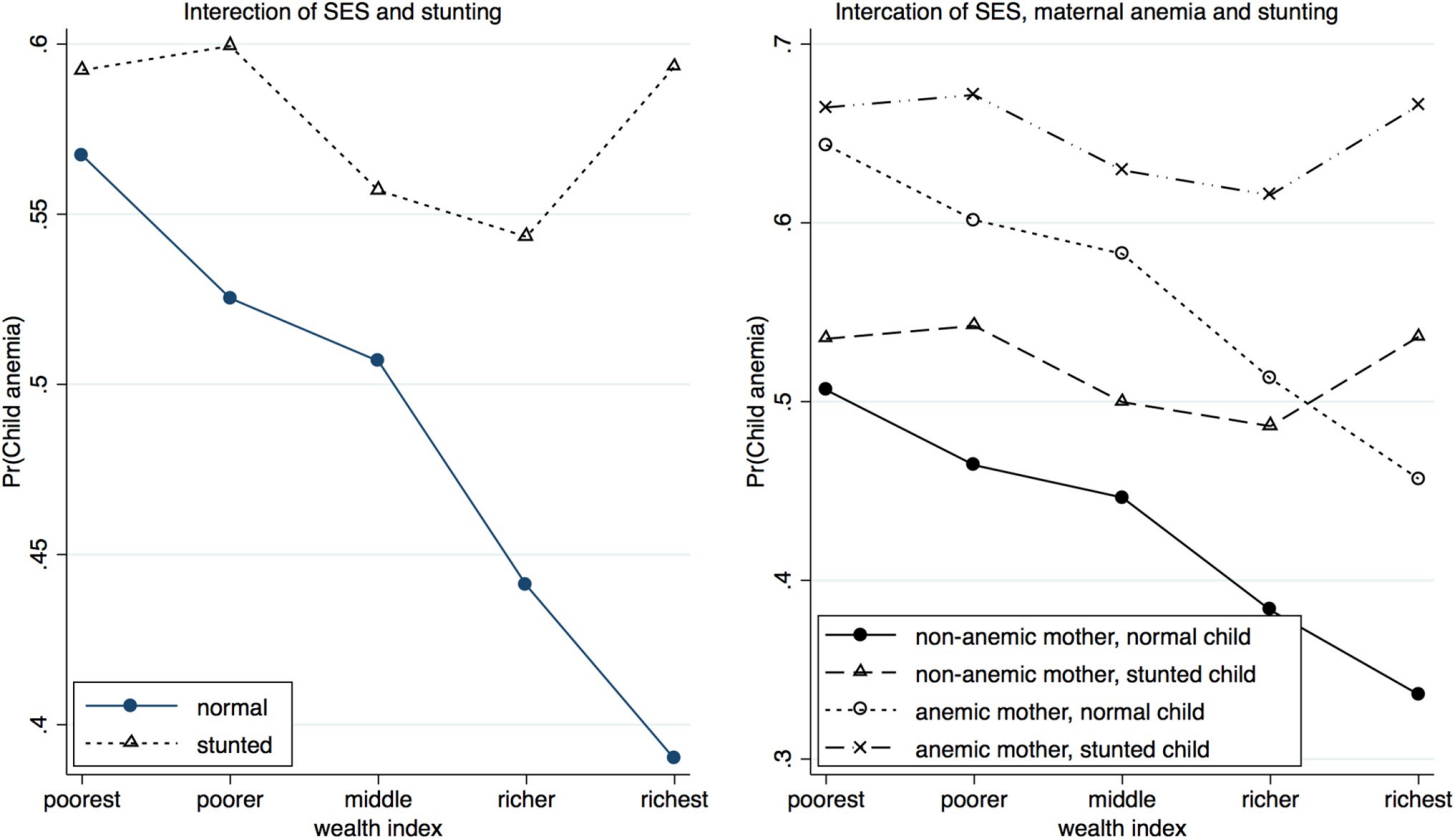
Interaction plots describing the association of stunting interact with SES, and maternal anemia, anemia separately on the risk of child anemia.

Further, the associations between stunting and anemia in children and low BMI and anemia in women were assessed by controlling for several confounders simultaneously in a multivariable binary logistic model, where confounders were selected based on p-value (<0.10) of the likelihood ratio test. In the final model for child anemia, the exposure stunting along with the covariate child’s age and its quadratic form, and the confounders maternal anemia and SES, and three—way interaction between stunting, SES and age were found to be significant (Table 5). The results suggest that stunted children are more likely to develop anemia compared to normal children with the estimated adjOR being 1.76 (95% CI: 1.10–2.83). The significant interaction effect of stunting with SES and age suggests that the effect of stunting could be modified by SES and age of the child. Fig 1 describes how stunting interacts with age and SES. It suggests that the likelihood of developing anemia decreases with increasing age and the rate of decrease is comparatively high till the age of 36 months. In addition, the risk of developing anemia decreases with the improvement of household economic condition, especially in the case of normal children. For stunted children, the decline is less. The differences in risk between stunted children and normal children at various ages are largest in the richest category, while the curves for the risk cross in the poorest category. SES is a strong modifier of the effect of stunting on anemia. Improvement in SES results in rapid decline in the risk of developing anemia among normal children but not in stunted children. (Fig 2: left). The effect of stunting also varies across the levels of maternal anemia and SES (Fig 2: right). The stunted children of anemic mothers are at greatest risk of developing anemia whereas the normal children of non-anemic mothers are at the lowest risk of developing anemia. The difference in risk between these groups increases slightly with increasing SES. Interestingly, non-stunted children of anemic mothers have elevated risk of developing anemia compared to non-stunted children of anemic mothers across all SES. Improvement in SES results in rapid decline in risk among normal children but not as much among stunted children. Thus, both maternal anemia and SES are strong effect modifiers of stunting.

**Table 5:** Results from multivariable logistic regression analysis of child anemia data.

Variables	OR	P-value	95% CI	
**Stunting**				
normal	RC			
stunted	1.763	<0.01	1.099	2.829
**Child age**	0.929	<0.001	0.902	0.956
**Child age^2**	1.003	<0.01	1.001	1.004
**maternal anemia**	1.801	<0.001	1.501	2.160
Stunting*SES*child age				
normal*poorest	RC			
normal*poorer	0.995	0.320	0.984	1.005
normal*middle	0.992	0.145	0.982	1.003
normal*richer	0.984	<0.01	0.974	0.994
normal*richest	0.976	<0.001	0.966	0.986
stunted*poorest	0.987	0.085	0.972	1.002
stunted*poorer	0.988	0.129	0.972	1.004
stunted*middle	0.982	<0.05	0.967	0.998
stunted*richer	0.981	<0.05	0.965	0.997
stunted*richest	0.987	0.150	0.970	1.005
Constant	4.347	<0.001	2.824	6.692

**Fig 3:**
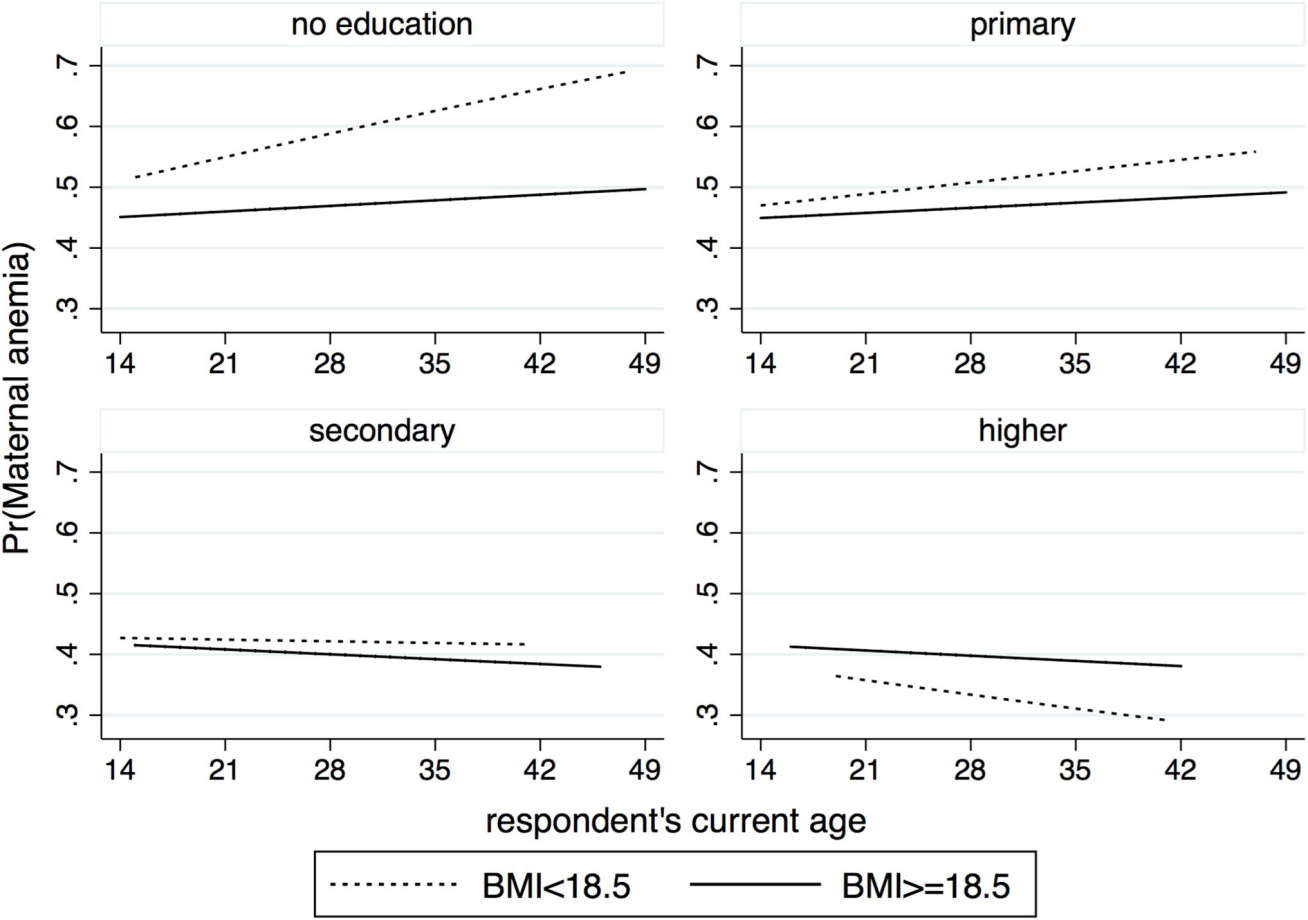
Interaction plot describing the association of BMI interact with education and age on the risk of maternal anemia.

**Fig 4:**
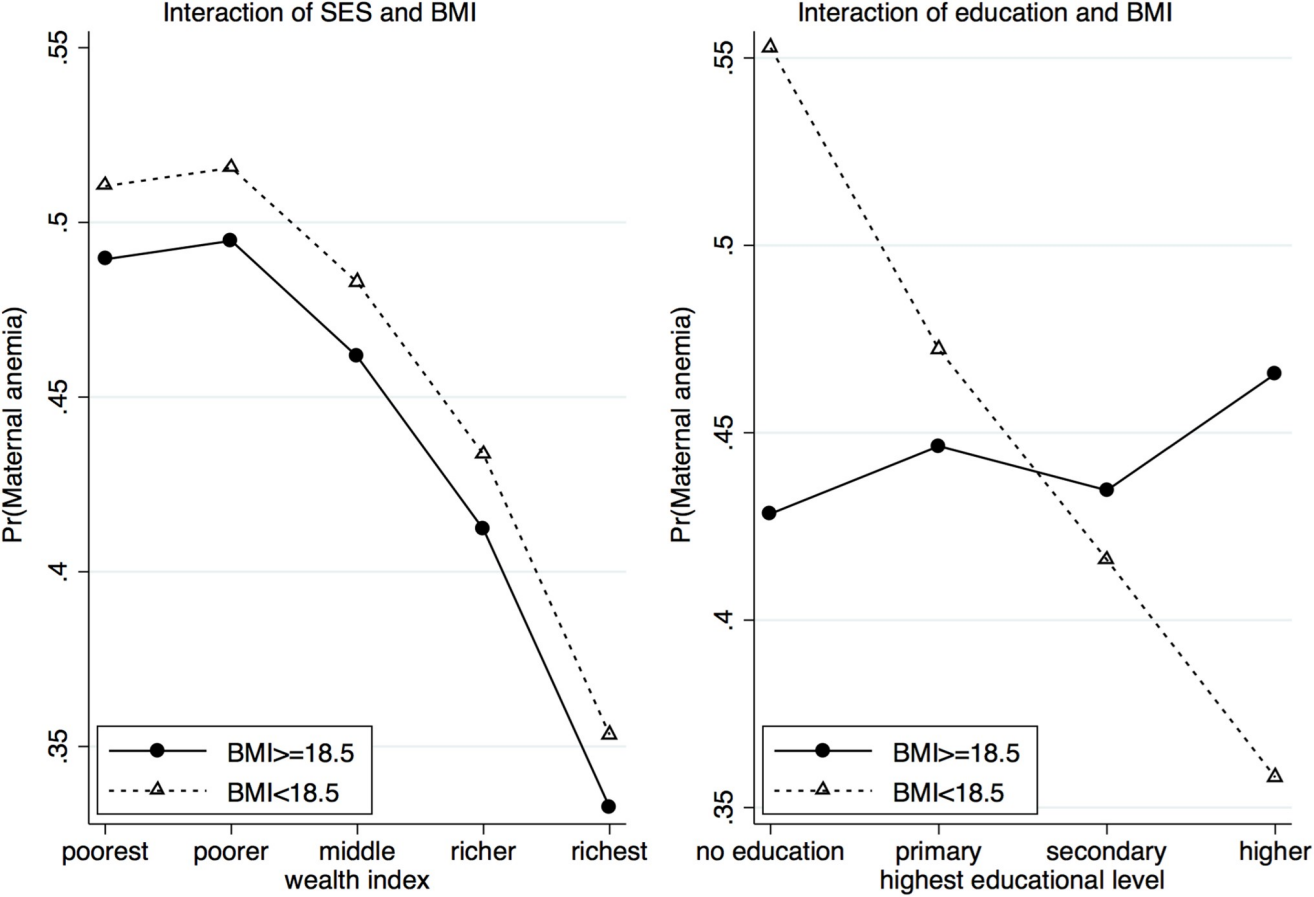
Interaction plot describing the association of BMI interact with SES and education, separately on the risk of maternal anemia.

Similarly, in the final multivariable model for women anemia, BMI is found to be significantly associated with anemia status, with the estimated adjOR of 1.80 (95% CI: 1.10–3.48) (Table 6). The interaction plot for BMI, age and education suggests that the risk of maternal anemia increases with increasing age for women with no education or primary education and decreases with age for women with secondary and higher education (Fig 3). In addition, the differences in risk at various ages between women with low BMI and women with normal BMI are significantly greater for uneducated women and diminish with increasing levels of education. Interestingly, for women with higher education, the risk of developing anemia is less in women with low BMI and the risk decreases with age. Fig 4 shows how SES and education modify the effect of BMI in women. The risk of being anemic decreases with increasing SES and the differences in risk between the two groups (low BMI and normal BMI) remain almost the same across all SES levels (Fig 4: left). Increasing education leads to significant drop in the risk of developing anemia among women with low BMI. In contrast, education does not appear to have any significant effect among women with normal BMI levels (Fig 4: right).

**Table 6:** Results from multivariable logistic regression analysis of maternal anemia data.

Variables	OR	P-value	95% CI	
BMI				
BMI>18.5				
BMI< = 18.5	1.807	<0.05	1.100	3.485
**SES**				
poorest	RC			
poorer	1.022	0.866	0.798	1.308
middle	0.894	0.395	0.690	1.157
richer	0.731	<0.05	0.560	0.953
richest	0.519	0.000	0.388	0.694
Education*BMI*age				
no educ*BMI> = 18.5	1.012	0.162	0.995	1.029
no educ*BMI<18.5	1.009	0.470	0.985	1.033
primary*BMI> = 18.5	1.015	0.111	0.997	1.034
primary*BMI<18.5	0.996	0.784	0.969	1.024
secondary*BMI> = 18.5	1.013	<0.05	1.011	1.034
secondary*BMI<18.5	0.987	0.411	0.958	1.018
higher*BMI> = 18.5	1.018	<0.05	1.012	1.040
higher*BMI<18.5	0.978	0.330	0.935	1.023
Constant	0.670	0.112	0.409	1.098

## Discussion

This paper has investigated the association between anemia and stunting among children in age group 6–59 months as well as the association between anemia and BMI among women of reproductive. In general, the prevalence of anemia was markedly high both among stunted children and women of low BMI compared to their normal counterparts. However, the higher prevalence among children suggests that they are more vulnerable to anemia than women. In both cases, there was a positive association between nutritional deficiency (reflected by stunting or low BMI) and anemia that was statistically significant even after controlling for the effects of possible confounders. This study identified important interactions that have interesting interpretations. There was significant interaction between stunting, child’s age and household socio-economic condition (SES). The risk of child anemia decreased with increasing age, however, the rate of decline was lower for stunted children. The implication of this finding is that there is a greater prevalence of anemia among stunted children compared to non-stunted children. In general, improvements in socio-economic status decreased the risk of being anemic in both groups. Thus, very young children belonging to poor households and experiencing stunting form a high risk group and should be the focus of interventions. Again, the presence of maternal anemia significantly increased the risk of child anemia even when there was improvement in socio-economic condition. The interaction effect of stunting and maternal anemia status suggests that stunted children of anemic mothers are at greater risk of being anemic. The strong association observed between maternal anemia and child anemia may be explained by the fact that there are certain factors influencing anemia that are common to both [43]. For example, both the mother and child could have a common dietary pattern and access to the same source of iron-rich micronutrient food. In addition, they share the same environment, have access to the same health facilities and are likely to have similar genetic traits. On the other hand BMI, which reflects nutritional deficiency among the women of reproductive age, is found to be significantly associated with maternal anemia. Although the risk of being anemic decreases with improvements in household economic condition, the difference in risk between women with low BMI and those with normal BMI remained almost the same. On the other hand, education strongly modifies the effect of BMI and has a profound effect on women with low BMI. Higher education lowers the risk of being anemic even if the BMI is low. These findings are similar to those found in other studies conducted for relevant population with similar [27, 28, 44] or different settings [29, 31, 43].

The results obtained in the study have some important implications. The strong associations between stunting and anemia in children, and low BMI and anemia in women, could be due to several factors. Nutritional deficiency may not be directly associated with anemia, however, it leads to certain changes in the body that make it susceptible to health hazards that may cause anemia. One hypothesis is that children and women suffering from nutritional deficiency are more likely to have weaker immune systems which make them vulnerable to various illnesses and health hazards such as parasitic infections or chronic inflammation [45–46]. Many of these conditions reduce the hemoglobin level in blood leading to increased anemia prevalence. The statement is supported by the evidence given in other studies [47] that nutritional deficiency causes several health hazards.

Strong associations between stunting and anemia in children as well as BMI and anemia in women indicate that it is necessary to tackle both nutritional deficiency and anemia simultaneously under the same program targeting both mother and children. Improving women’s education and empowerment might be one way to curb high anemia and malnutrition prevalence rates. Another possible tool to tackle anemia and malnutrition is mass-and-social-media campaign which works by increasing awareness and knowledge about these conditions and methods of prevention.

### Strengths and limitations of the study

This study is based on data from a nationally representative survey, which is regularly conducted by an international expert group, and hence the quality of data is high. In addition, findings from nationally representative data are more helpful for policy makers to design appropriate interventions. Furthermore, based on data conditions, sophisticated epidemiological and statistical analyses have been performed to meet the main objective of the study.

However, this study has some limitations. These include i) unavailability of data on intake of iron rich food for all subjects [37], ii) data may suffer from re-call bias of information on SES, size of the child at birth and evidence of fever or diarrhea in last fifteen days of the survey, and iii) measurement errors in data on anemia (Hb level).
